# Supplementary material for: Spin environment of a superconducting qubit in high magnetic fields
Source: Nat Commun. 2025 Oct 29;16:9564. doi: 10.1038/s41467-025-65528-y (PMC12572138; doi:10.1038/s41467-025-65528-y)
Supplement: Supplementary file 1 — Supplementary Information [file 41467_2025_65528_MOESM1_ESM.pdf]

Supplementary Information for
Spin Environment of a Superconducting Qubit in High Magnetic Fields

S. Günzler,[1,2,*] J. Beck,[1,†] D. Rieger,[1] N. Gosling,[2] N. Zapata,[2] M. Field,[2] S. Geisert,[2]
A. Bacher,[3,4] J. K. Hohmann,[3,4] M. Spiecker,[1,2] W. Wernsdorfer,[1,2] and I. M. Pop[1,2,5,‡]

[1] *PHI, Karlsruhe Institute of Technology, 76131 Karlsruhe, Germany*
[2] *IQMT, Karlsruhe Institute of Technology, 76131 Karlsruhe, Germany*
[3] *IMT, Karlsruhe Institute of Technology, 76131 Karlsruhe, Germany*
[4] *KNMFi, Karlsruhe Institute of Technology, 76131 Karlsruhe, Germany*
[5] *Physics Institute 1, Stuttgart University, 70569 Stuttgart, Germany*

CONTENTS

I. Sample Holder and Qubit Measurement 2

II. Flux Sensitivity in Gradiometric and Non-Gradiometric Gralmonium Qubits 3

III. Flux Noise in Magnetic Field 3

IV. TLS Hyperpolarization 4

References 5

* simon.guenzler@kit.edu

† First two authors contributed equally.

‡ ioan.pop@kit.edu

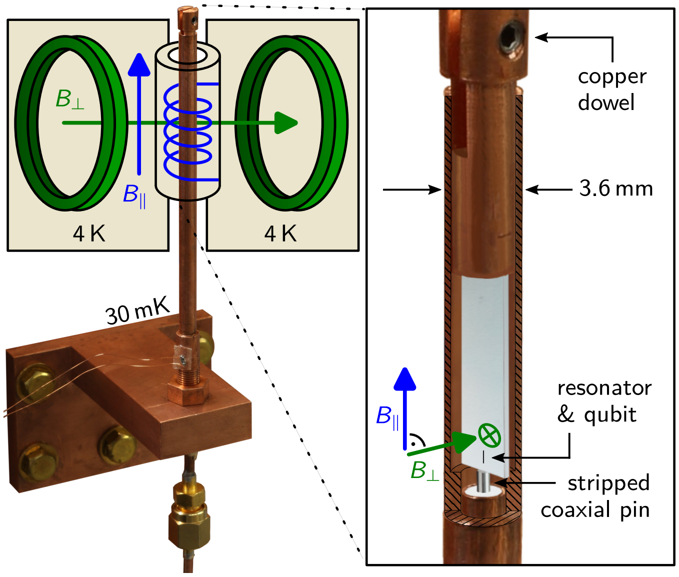

FIG. S1. **Cylindrical waveguide sample holder within the vector magnet.** The 2D vector magnet is thermalized on the 4 K stage of the cryostat and separated by a 1 mm gap from the cylindrical pipe of the sample holder, which is anchored to the 30 mK stage. The waveguide, with a 3 mm inner diameter and 0.3 mm wall thickness, has a cut-off frequency of ~ 60 GHz, operating in the sub-wavelength regime. This results in coupling via the evanescent microwave field of a stripped coaxial pin, with an exponential decay in coupling strength relative to the chip-to-pin distance (cf. [1, 2]). The circuit is positioned on the bottom of a 3 mm x 10 mm sapphire chip and fixed with a copper dowel, clamped against the cylindrical copper pipe walls. Apiezon N vacuum grease on the dowel provides additional thermal anchoring. High magnetic fields are applied in the substrate plane via a solenoid coil, while magnetic flux tuning is achieved with a Helmholtz pair aligned perpendicular to the substrate plane. No additional shielding is implemented between the sample holder and vector magnet coils. Note that the setup is identical to the one used in Ref. [3].

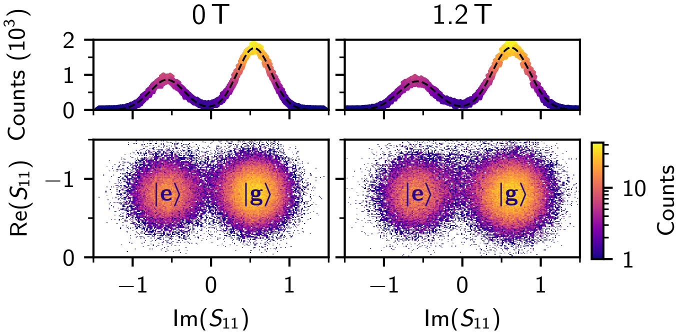

FIG. S2. **IQ histogram in magnetic field:** 1D histogram of the measured I quadrature (top panels) and 2D histogram of the I and Q quadrature (bottom panels). In $B_\parallel = 0$ T (left panels) and $B_\parallel = 1.2$ T (right panels), we extract a qubit population corresponding to a temperature of 165 mK and 150 mK, respectively. These IQ histograms are illustrative for all qubit state measurements used in the main text. We use a 540 ns readout pulse with an equilibrium average photon number of $\bar{n} = 25$, integrated over 540 ns, including resonator ring-up and ring-down times corresponding to a linewidth $\kappa/2\pi = 1.2$ MHz.

I. SAMPLE HOLDER AND QUBIT MEASUREMENT

In Fig. S1, we present the readout and thermalization of our sample housed within a copper waveguide sample holder (design identical to Refs. [1–3]). The sample is thermally anchored to the 30 mK stage of the cryostat by clamping it with a copper dowel into the sample holder and applying Apiezon N vacuum grease for additional thermal contact. The readout resonator, which we read out in single-port reflection, is positioned in close proximity to the stripped pin of a coaxial cable to couple to its evanescent microwave field. The 2D vector magnet is centered on the resonator and attached to the 4 K stage of the cryostat. We attribute the high thermal population of $p_{\mathrm{th}} \sim 40\,\%$ at a qubit frequency of 2.36 GHz (cf. Fig. 3) to thermal photons leaking into the sample holder due to the absence of additional thermal shielding. In contrast, a similar device in the same sample holder geometry was reported in Ref. [2] with a qubit temperature of 37 mK, where improved IR shielding and thermalization was possible due to the absence of the vector magnet. In Fig. S2, we compare measured IQ histograms in $B_\parallel = 0$ T and $B_\parallel = 1.2$ T and we find similar qubit populations and signal to noise ratio. To reduce thermal photon infiltration via the microwave lines, an infrared filter is placed in front of the sample holder. From our setup we can identify potential culprits for increased magnetic field noise, such as vibrations of the cylindrical waveguide sample holder within the vector magnet, fluctuations in the vector magnet power supply or vortex retrapping within the coil.

To avoid a hysteretic, non-monotonic dependence of the resonator response on magnetic field, we apply high magnetic fields only in the substrate plane ($B_\parallel$) and minimize the out-of-plane component ($B_\perp$). We account for minor chip misalignments, including tilt and rotation, by determining a compensation field $B_{\perp,\mathrm{comp}}$ for each $B_\parallel$, ensuring that all qubit measurements are conducted within the flux period closest to zero effective out-of-plane field. Out-of-plane magnetic fields induce screening currents in the resonator that suppress the resonance frequency. We sweep $B_\perp$ and find the compensation field by maximizing the resonator frequency. We determine an effective chip misalignment of 0.66 mT/T. A detailed description of this compensation procedure can be found in the supplementary information of Ref. [3].

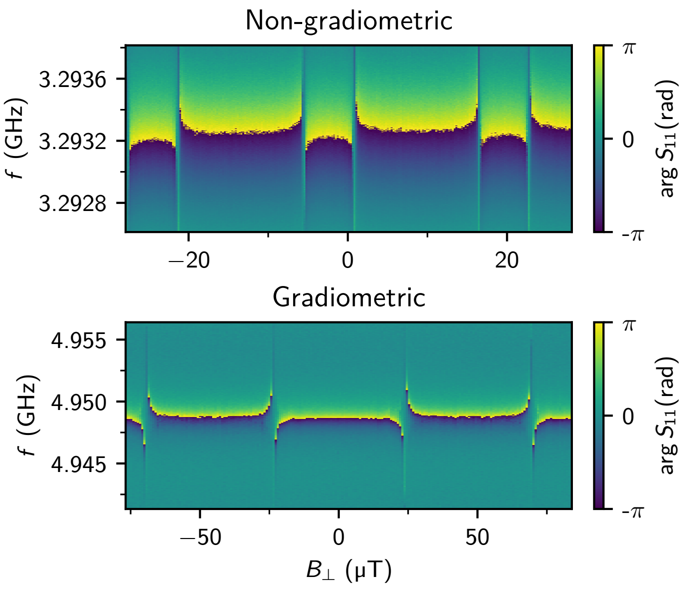

FIG. S3. **Flux sensitivity in a gradiometric and non-gradiometric gralmonium.** A flux sweep of the resonator phase response $\arg(S_{11})$ for non-gradiometric (top panel) and gradiometric (bottom panel) gralmonium qubits shows avoided level crossings where the qubit frequency intersects the resonator frequency. We observe a 4.6-fold increase in flux periodicity from the non-gradiometric to the gradiometric qubit design.

II. FLUX SENSITIVITY IN GRADIOMETRIC AND NON-GRADIOMETRIC GRALMONIUM QUBITS

In addition to the gradiometric qubit design shown in Fig. 1a, we fabricate a non-gradiometric fluxonium qubit with a similar layout: Here, we removed the connecting wire that would close the second flux loop ($\Phi_{\mathrm{ext},2}$), resulting in a gralmonium with only one flux loop, illustrated in violet in Fig. 1a ($\Phi_{\mathrm{ext}} = \Phi_{\mathrm{ext},1}$, the inductance L_3 in Fig. 1b is removed). In Fig. S3, we compare the flux periodicities of the gradiometric and non-gradiometric design, finding an increase by a factor of 4.6. This increase is consistent with the flux loop size ratio $\Phi_{\mathrm{ext},1}/\Phi_\Delta$, suggesting negligible inductance asymmetry $\alpha \approx 0$. Note that in our qubit implementation, one flux loop extends into the resonator, where the average screening current flows along the center of the 4 µm-wide strip due to the high impedance of the resonator, as illustrated in Fig. 1a.

III. FLUX NOISE IN MAGNETIC FIELD

Figure S4 illustrates the fitting procedure for Hahn echo decay measurements as a function of external flux. At the half-flux sweet spot ($\Phi_{\mathrm{ext}}/\Phi_0 = 0.5$), the decay is purely exponential, while deviations from this point ($\Phi_{\mathrm{ext}}/\Phi_0 \neq 0.5$) reveal an additional Gaussian dephasing component, $\Gamma^{\Phi}_{\varphi\mathrm{E}}$. The echo decay is modeled as [4]

$$P_{|e\rangle}(t) = \frac{1}{2} e^{-(\Gamma^{\Phi}_{\varphi\mathrm{E}} t)^2} \cdot e^{-(\Gamma_1/2+\Gamma^{\mathrm{const}}_{\varphi\mathrm{E}})t} + \frac{1}{2}\,. \tag{S1}$$

The exponential decay rate $\Gamma_{\mathrm{exp}} = \Gamma_1/2 + \Gamma^{\mathrm{const}}_{\varphi\mathrm{E}}$, combines energy relaxation and exponential dephasing, both assumed to be flux-independent over the range $\Phi_{\mathrm{ext}} = 0.48$–$0.52\,\Phi_0$. Accordingly, we perform a joint fit for every set of Hahn echo measurements vs. flux, extracting the flux-dependent Gaussian dephasing rate $\Gamma^{\Phi}_{\varphi\mathrm{E}}$ atop the fixed exponential decay envelope defined by Γ_{exp}.

In Fig. S5, we report the magnetic field dependence of the flux noise amplitude for three qubit frequencies at half flux bias. We obtain the change in the qubit frequency at half flux bias by thermal cycling of our device to room temperature. We observe a suppression of the flux noise amplitude across all qubit frequencies, which we model using two-level fluctuators generating asymmetric random telegraph signals with corresponding Lorentzian power spectrum

$$S(\omega) \propto \frac{1}{\Gamma_1/\Gamma_\uparrow + \Gamma_1/\Gamma_\downarrow} \frac{\Gamma_1}{\Gamma_1^2+\omega^2}\,. \tag{S2}$$

We assume the total decay rate Γ_1 remains invariant under magnetic field. To rewrite the amplitude of the power spectrum in Eq. (S2) we use detailed balance $\dot{p_0} = \Gamma_\downarrow p_1 - \Gamma_\uparrow p_0$ where p_0 and p_1 are the population probabilities for the ground and excited state, respectively. At thermal equilibrium, $\dot{p_0} = 0$, we obtain

$$\Gamma_\uparrow = \Gamma_1 p_{\mathrm{th}} \tag{S3}$$
$$\Gamma_\downarrow = \Gamma_1(1-p_{\mathrm{th}})\,, \tag{S4}$$

where p_{th} is the excited state population probability in thermal equilibrium. Assuming Boltzmann-distributed populations

$$\frac{p_1}{p_0} = \frac{p_{\mathrm{th}}}{1-p_{\mathrm{th}}} = e^{\frac{-\Delta E}{k_{\mathrm{B}}T}}\,, \tag{S5}$$

with the energy difference ΔE between the ground and excited state, the flux noise power becomes

$$1/A_\Phi \propto \frac{\Gamma_1}{\Gamma_\uparrow} + \frac{\Gamma_1}{\Gamma_\downarrow} \tag{S6}$$
$$= \frac{1}{p_{\mathrm{th}}} + \frac{1}{(1-p_{\mathrm{th}})} \tag{S7}$$
$$= \left(e^{\frac{-\Delta E}{k_{\mathrm{B}}T}}+1\right)\left(e^{\frac{\Delta E}{k_{\mathrm{B}}T}}+1\right) \tag{S8}$$
$$= 4\cosh^2\left(\frac{\Delta E}{2k_{\mathrm{B}}T}\right) \tag{S9}$$
$$\sqrt{A_\Phi} \propto \frac{1}{\cosh\left(\frac{\mu_{\mathrm{B}}B}{k_{\mathrm{B}}T}\right)}\,, \tag{S10}$$

where we inserted the energy difference $2\mu_{\mathrm{B}}B$ of $g = 2$ spin $s = 1/2$ paramagnetic impurities in the last step.

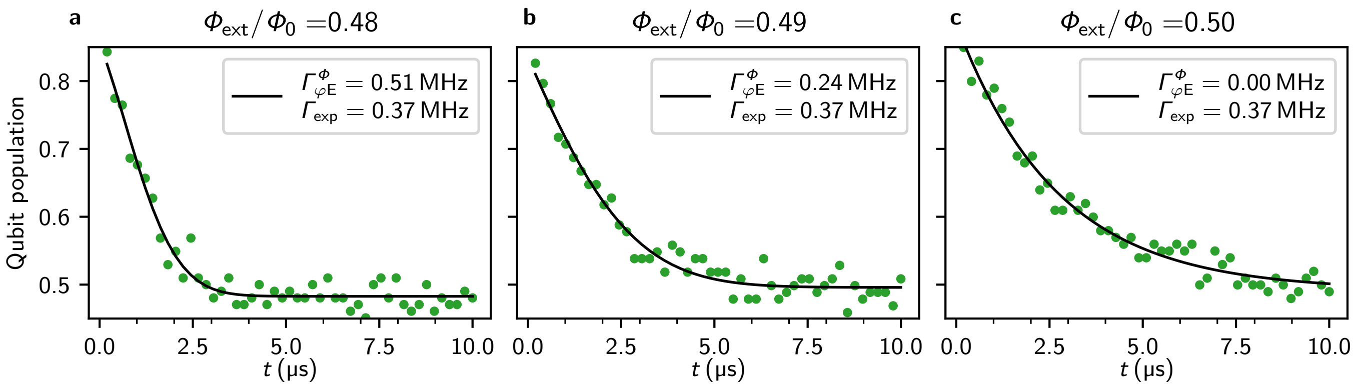

FIG. S4. **Flux-dependence of the Hahn echo decay.** Echo measurement with a single refocusing π-pulse at three representative flux biases: **a** $\Phi_{ext}/\Phi_0 = 0.48$, **b** $\Phi_{ext}/\Phi_0 = 0.49$ and **c** $\Phi_{ext}/\Phi_0 = 0.5$. Black lines indicate a joint fit to 42 individual echo measurements acquired over the flux range $\Phi_{ext} = 0.48$–$0.52\,\Phi_0$, using Eq. (S1), which includes a constant exponential decay rate Γ_{exp} and a flux-dependent Gaussian dephasing contribution $\Gamma^{\Phi}_{\varphi E}$.

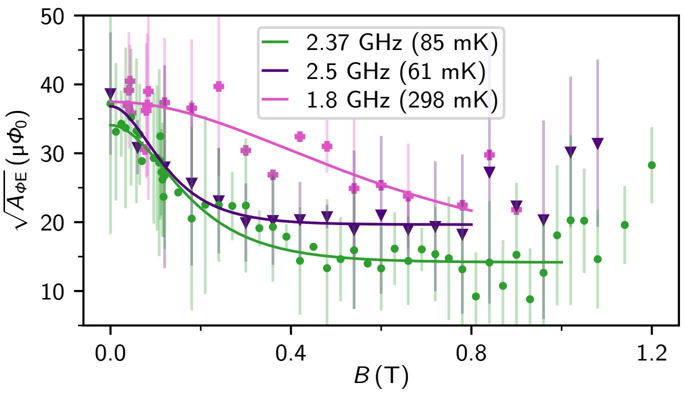

FIG. S5. **Flux noise in magnetic field.** The flux noise amplitude from echo experiments (cf. Eq. 3) decreases in magnetic field. Different colors and marker shapes correspond to different qubit frequencies at half flux bias, obtained in different cooldowns of the same device. Lines show fits to Eq. 4. Marker shapes are identical to the inset in Fig. 2d.

Fits to Eq. (S10) are in good agreement with the measured flux noise amplitudes (cf. Fig. S5). The observed variation in extracted spin bath temperatures motivates further noise characterization, including measurements at different cryostat temperatures. To distinguish between various sources of flux-bias-dependent noise, such as flux noise from the spins, vortex noise in the coil, or kinetic inductance fluctuations in the qubit superinductor, a comprehensive analysis of the qubit power spectral density is required, similar to Ref. [5].

IV. TLS HYPERPOLARIZATION

In Fig. S6 we present the TLS hyperpolarization experiment for a varying number $N = 1$–10^4 of active qubit state preparations within the stabilization sequence. We use an active qubit reset sequence, i.e. a readout followed by a conditional π-pulse which prepares the qubit in the target state ($|e\rangle$ or $|g\rangle$). This feedback-based stabilization is repeated N times (cf. Fig. 3a). The cross-relaxation rate between the qubit and a TLS labeled by index k is described by [6, 7]

$$\Gamma^k_{\mathrm{qt}} = \frac{2g^2\Gamma_2}{\Gamma_2^2 + \delta_k^2}\,, \tag{S11}$$

where g is the qubit-TLS coupling strength, Γ_2 is the total decoherence rate of the coupled system, and δ_k is the frequency detuning between the qubit and the k-th TLS. Through repeated stabilization, the TLSs are polarized towards the qubit stabilization state via the cross-relaxation (Eq. (S11)), resulting in significant population transfer. For stabilization in $|e\rangle$, this induces a population inversion (hyperpolarization) of the TLSs, exceeding the thermal limit of 50%, similar to spectral hole burning.

To model the dynamics, following Ref. [6] we consider a qubit coupled to a frequency-distributed ensemble of TLSs, governed by the Solomon rate equations [7]:

$$\dot{p}_{\mathrm{q}} = -\Gamma_{\mathrm{q}}(p_{\mathrm{q}} - p_{\mathrm{th}}) - \sum_k \Gamma^k_{\mathrm{qt}}(p_{\mathrm{q}} - p^k_{\mathrm{t}}) \tag{S12}$$

$$\dot{p}^k_{\mathrm{t}} = -\Gamma^k_{\mathrm{t}}(p^k_{\mathrm{t}} - p_{\mathrm{th}}) - \Gamma^k_{\mathrm{qt}}(p^k_{\mathrm{t}} - p_{\mathrm{q}}), \tag{S13}$$

where p_{q} and p^k_{t} are the qubit and TLS populations, Γ_{q} and Γ^k_{t} are their respective intrinsic relaxation rates, and p_{th} is the thermal equilibrium population. To reduce model complexity, following Ref. [6], we assume TLSs are equally spaced in frequency, $\delta_k = k\Delta + \Delta_0$, with identical g and Γ_2. Here, Δ is the inter-TLS spacing, and $\Delta_0 \in [0, \Delta/2]$ represents a frequency offset relative to the qubit. The data in Fig. S6 are jointly fitted using this simplified model, yielding a total cross-relaxation $\sum \Gamma_{\mathrm{qt}} = 45\,\mathrm{kHz}$ and an intrinsic qubit relaxation rate $\Gamma_q = 140\,\mathrm{kHz}$.

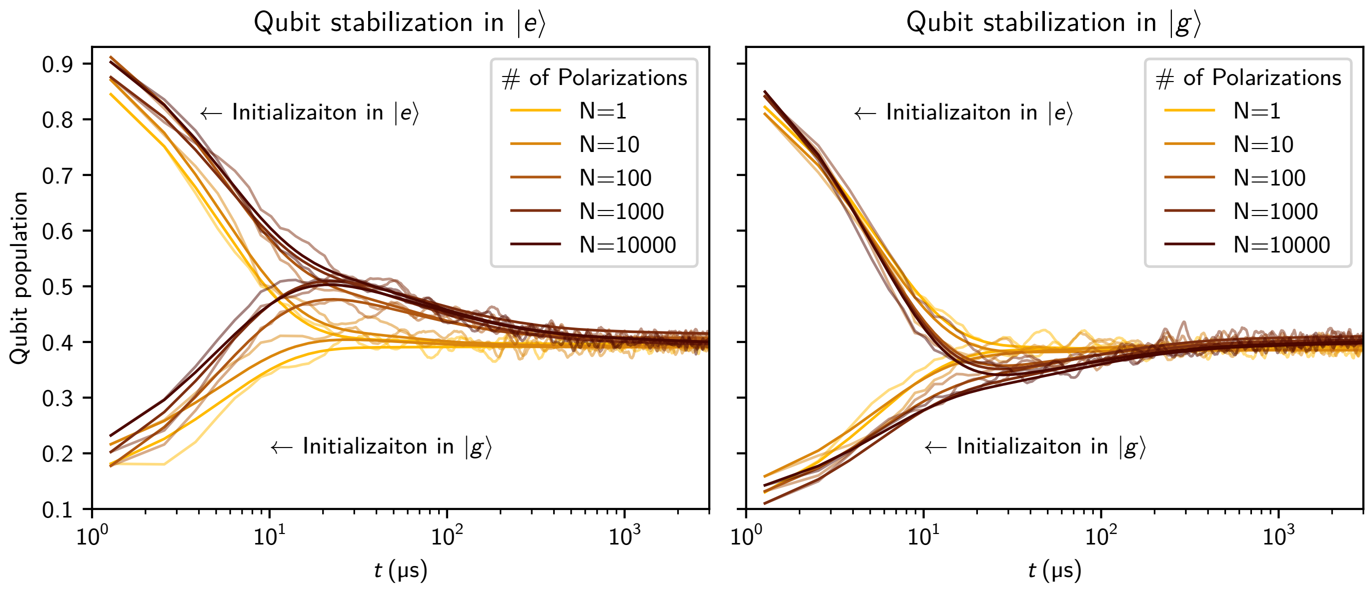

FIG. S6. **TLS hyperpolarization experiment with varying numbers of qubit preparations in the stabilization sequence.** Qubit population relaxation, measured stroboscopically at intervals of $\delta t = 1.28\,\mu\text{s}$ following the preparation sequence shown in Fig. 3a. The sequence consists of N active state preparations in either $|e\rangle$ (left panel) or $|g\rangle$ (right panel), followed by qubit initialization in $|g\rangle$ or $|e\rangle$. Semi-transparent lines show linearly sampled data on a logarithmic axis, smoothed using a Savitzky-Golay filter with a window length scaling with the square root of the data index. Opaque lines represent a joint fit of the full data set to the theoretical model described in Refs. [6, 7] (see main text). Note that the data for $N = 10000$ is identical to that in Fig. 3b in the main text.

[1] D. Rieger, S. Günzler, M. Spiecker, A. Nambisan, W. Wernsdorfer, and I. M. Pop, Fano Interference in Microwave Resonator Measurements, Phys. Rev. Appl. **20**, 014059 (2023).

[2] D. Rieger, S. Günzler, M. Spiecker, P. Paluch, P. Winkel, L. Hahn, J. K. Hohmann, A. Bacher, W. Wernsdorfer, and I. M. Pop, Granular aluminium nanojunction fluxonium qubit, Nat. Mater. **22**, 194 (2023).

[3] K. Borisov, D. Rieger, P. Winkel, F. Henriques, F. Valenti, A. Ionita, M. Wessbecher, M. Spiecker, D. Gusenkova, I. M. Pop, and W. Wernsdorfer, Superconducting granular aluminum resonators resilient to magnetic fields up to 1 Tesla, Appl. Phys. Lett. **117**, 10.1063/5.0018012 (2020).

[4] J. Schriefl, Y. Makhlin, A. Shnirman, and G. Schön, Decoherence from ensembles of two-level fluctuators, New J. Phys. **8**, 1 (2006).

[5] F. Yan, S. Gustavsson, A. Kamal, J. Birenbaum, A. P. Sears, D. Hover, T. J. Gudmundsen, D. Rosenberg, G. Samach, S. Weber, J. L. Yoder, T. P. Orlando, J. Clarke, A. J. Kerman, and W. D. Oliver, The flux qubit revisited to enhance coherence and reproducibility, Nat. Commun. **7**, 1 (2016).

[6] M. Spiecker, P. Paluch, N. Gosling, N. Drucker, S. Matityahu, D. Gusenkova, S. Günzler, D. Rieger, I. Takmakov, F. Valenti, P. Winkel, R. Gebauer, O. Sander, G. Catelani, A. Shnirman, A. V. Ustinov, W. Wernsdorfer, Y. Cohen, and I. M. Pop, Two-level system hyperpolarization using a quantum Szilard engine, Nat. Phys. **19**, 1320 (2023).

[7] M. Spiecker, A. I. Pavlov, A. Shnirman, and I. M. Pop, Solomon equations for qubit and two-level systems: Insights into non-Poissonian quantum jumps, Phys. Rev. A **109**, 052218 (2024).
